# Eichorst’s Practice of Medicine

**Published:** 1901-08

**Authors:** 


EICHORST'S PRACTICE OF MEDICINE. A text-book of the practice of medicine. By Dr. Herman Eichorst, Professor of Special Pathology and Therapeutics and Director of the Medical Clinic in the University of Zurich. Translated and edited by Augustus A. Eshner, M. D., Professor of Clinical Medicine in the Philadelphia Polyclinic. Two octavo columes of over 600 pages each; over 150 illustrations. Philadelphia and London: W. B. Saunders & Co. 1901. Price per set, cloth, $6.00 net.

The author is eminently qualified to write a text-book on the practice of medicine, having been for many years one of the most distinguished of the German clinicians and writers on medical subjects. His connection with the Zurich University Clinic has afforded him an unusually good opportunity for the study of disease as it is met with in general practice. Most of the new smaller text-books and manuals on the practice of medicine are mere compilations, done by men of ordinary ability and of limited experience as observers. To such books Dr. Eichorst's masterly work presents a striking contrast. Throughout it bears evidence that the author has gained much of his knowledge of the general symptomatology and management of disease "first hand." In almost every instance he is able to recommend the plan of treatment which his own experience has demonstrated to be best. However, other lines of treatment in good repute with the profession are given due attention. The descriptions of comparatively unimportant diseases are very much condensed, thus room is left for a fuller discussion of the more important ones. Most of the illustrations, of which there are a goodly number, are made from photographs taken of cases personally observed by the author.

The important points in diagnosis and treatment are everywhere properly emphasized by a judicious use of heavy type and italics.

As a reliable and practical guide for the general practitioner and the student we do not believe this work has many equals in the English language at least.

